# Innovative Categorization and Operative Management of Auditory Ossicle Disruption Following Trauma: Therapeutic Efficacy and Interventional Paradigms

**DOI:** 10.7150/ijms.103532

**Published:** 2024-10-14

**Authors:** Yajing Wang, Wenjun Chen, Jiahao Liu, Guowang Wang, Yongkang Ou

**Affiliations:** 1 Department of Otolaryngology, Head and Neck Surgery, Sun Yat-sen Memorial Hospital, Sun Yat-sen University, Guangzhou 510120, P.R. China.; 2 Department of Otolaryngology, Head and Neck Surgery, ShenShan Medical Center, Memorial Hospital of Sun Yat-sen University, Shanwei 516400, P.R. China.

**Keywords:** Traumatic Ossicular Chain Dislocation, Classification, Ossiculoplasty, Stapediovestibular Dislocation, Pneumolabyrinth

## Abstract

Currently, there is no consensus on the treatment protocol for ossicular chain trauma. This study aims to investigate the classification and treatment strategies for traumatic ossicular chain dislocation. We retrospectively analyzed 15 patients. Traumatic ossicular chain dislocations were categorized based on the location of trauma identified during surgery: Type I—ossicular trauma without stapediovestibular dislocation; Type II—stapediovestibular dislocation (with or without associated incus dislocation). Of the 10 patients with Type I trauma, 9 experienced head trauma, and 1 had a penetrating injury to the external auditory canal. Among these, 2 cases involved incudomalleolar dislocation, 2 cases incus dislocation, 5 cases incudostapedial dislocation, and 1 case a fracture of the anterior and posterior arches of the stapes. Seven patients exhibited conductive hearing loss, while 3 presented with mixed hearing loss. Ossiculoplasty was performed using partial ossicular replacement prostheses (PORP) in 8 patients and total ossicular replacement prostheses (TORP) in 2 patients. Postoperative air conduction thresholds significantly improved in all 10 patients. In Type II trauma, all 5 patients had a penetrating injury to the external auditory canal, resulting in varying degrees of hearing loss. Postoperatively, 3 patients experienced improvement in hearing, while 2 showed no significant change. All patients developed vertigo and tinnitus following the trauma, with vertigo resolving after surgery. Pneumolabyrinth was detected in 2 patients. We propose a novel classification system for traumatic ossicular chain dislocation. Treatment strategies should be tailored according to the specific trauma type.

## Introduction

Ossicular chain trauma can arise from various etiologies 1, 2, including head injuries, such as those resulting from traffic accidents or falls from heights, as well as penetrating injuries to the external auditory canal, often caused by the insertion of foreign objects. Head trauma is frequently accompanied by temporal bone fractures, leading to varying degrees of facial paralysis, whereas penetrating injuries typically do not result in such complications. Studies have suggested that head trauma is the most common cause of ossicular chain damage 3, 4. Hough 5 proposed three primary mechanisms underlying ossicular chain injury: 1) The shock from head trauma can cause skull fractures, leading to the instantaneous separation of tissues, thereby reducing their elasticity and structural integrity; 2) According to Newton's first law—which states that an object in motion or at rest will remain in that state unless acted upon by an external force—when the head is struck while relatively stationary, it moves, but the intracranial structures, including the ossicular chain, remain stationary, resulting in inertial forces that can cause injury to the ossicles; 3) Violent stimulation can cause the contraction of intratympanic muscles, subjecting the auditory ossicles to multidirectional forces 6.

Ossicular chain trauma typically manifests as either ossicular dislocation or fracture, with dislocation being more common. Research has shown that the incus is more frequently affected than the malleus and stapes 7-10. The difference in incidence may be due to the anatomical characteristics of each ossicle, rather than trauma alone. The stapes are protected by the annular ligament and the stapedius muscle, while the malleus is stabilized by the anterior and lateral ligaments, along with the tensor tympani tendon attached to the superior part of the malleus handle 11. Consequently, when the middle ear is subjected to trauma, the incus is more prone to displacement or fracture. Dislocation of the incus can occur at the incudomalleolar joint, the incudostapedial joint, or both, with incudostapedial dislocation being the most common 3.

Currently, the types of ossicular chain trauma have yet to be systematically classified. Clinically, ossicular chain disruption can be roughly categorized based on tympanic exploration into several types 3, 12, 13: incudostapedial dislocation, incudomalleolar separation or dislocation, dislocation of the malleus or incus, stapediovestibular dislocation (both external and internal), and ossicle fracture 14.

Due to the limited number of cases, particularly those involving stapediovestibular dislocation, no standardized treatment protocol currently exists. Given the relative lack of research, the purpose of this study is to investigate the classification and treatment of traumatic ossicular chain dislocation 15.

## Materials and Methods

### Ethical statement

All of the participants provided informed consent and the study received approval from the Institutional Review Board (SYSKY-2024-889-01) of Sun Yat-sen Memorial Hospital's ethics committee. Written informed consent was obtained from all participants in accordance with the principles defined in the Declaration of Helsinki.

### Study design

This retrospective study analyzed 15 patients diagnosed with traumatic ossicular chain dislocation at the Department of Otorhinolaryngology, Sun Yat-sen Memorial Hospital, Sun Yat-sen University, between 2015 and 2023. The patients' ages ranged from 14 to 62 years, with 11 males and 4 females. The etiologies of trauma included head trauma in 9 patients and penetrating injuries to the external auditory canal in 6 patients. One patient was admitted directly to the emergency department, while the remaining patients received initial conservative treatment at local hospitals. The interval between the trauma and admission to our hospital varied from 1 month to 6 years. The primary symptoms reported were hearing loss, vertigo, and tinnitus, as detailed in Table 1.

All patients underwent pure tone audiometry both preoperatively and 6 months postoperatively. Air conduction (AC) and air-bone gap (ABG) thresholds were calculated as averages across frequencies of 0.5, 1.0, 2.0, and 4.0 kHz. Additionally, all patients received high-resolution computed tomography (HRCT) scans of the temporal bone prior to surgery, with cranial MRI performed to exclude craniocerebral or cranial nerve injuries 16. Based on the intraoperative findings regarding the trauma location, traumatic ossicular chain dislocations were classified as follows: Type I, Ossicular trauma without stapediovestibular dislocation and Type II, Stapediovestibular dislocation (with or without associated incus dislocation).

### Statistical analysis

Statistical analysis was carried out with SPSS version 27.0 (IBM Corporation, NY, USA). Descriptive statistics were shown as mean ± standard deviation or median (inter-quartile range), based on the normality of the data. Independent t-tests or Mann-Whitney U tests were used for comparing groups, as suitable. Categorical data were presented as counts and percentages. For comparing categorical data between groups, Fisher's exact tests or chi-squared tests were applied. A two-sided p-value of less than 0.05 was considered statistically significant.

## Results

Table 1 provides a detailed overview of the clinical and treatment characteristics of the 15 patients included in this study. All patients exhibited varying degrees of hearing loss, with 8 patients (53.3%) presenting with conductive hearing loss, 6 patients (40%) with mixed hearing loss, and 1 patient (6.7%) with profound hearing loss. Additionally, 9 patients (60%) reported tinnitus, and 5 patients (33.3%) experienced vertigo.

**Figure 1:**
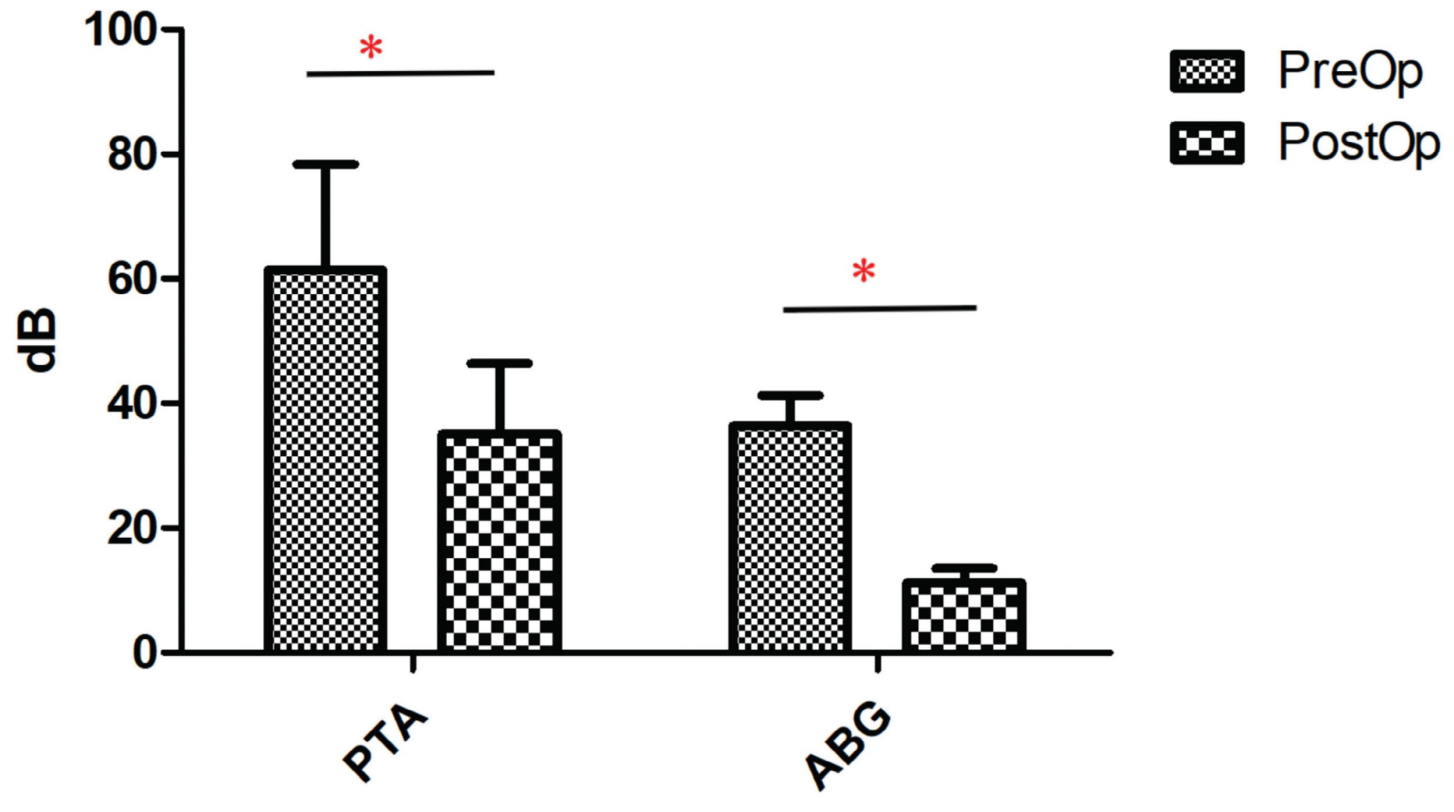
Comparison of pure-tone audiometry (PTA) and ABG thresholds of type I trauma patients before and after surgery. *p<0.05.

The traumas were categorized into two types based on the presence of stapediovestibular dislocation (as detailed in Table 2). Among the 10 patients classified with Type I trauma, 9 had sustained head trauma, while 1 had suffered a penetrating injury to the external auditory canal (caused by an ear pick). In this group, 2 patients exhibited incudomalleolar dislocation, 2 patients had incus dislocation (one of whom also had a fracture of the anterior and posterior arches of the stapes), 5 patients presented with incudostapedial dislocation, and 1 patient had a fracture of the anterior and posterior arches of the stapes. Of the 10 patients with Type I trauma, 7 had conductive hearing loss, and 3 had mixed hearing loss. Ossicular reconstruction was performed using a partial ossicular replacement prosthesis (PORP) in 8 patients and a total ossicular replacement prosthesis (TORP) in 2 patients. Postoperative pure tone audiometry revealed a significant improvement in hearing thresholds in all 10 patients (p < 0.05) (Figure 1).

All 5 patients with Type II trauma had sustained penetrating injuries, leading to varying degrees of hearing loss. Specifically, 2 patients had mixed hearing loss, 1 had conductive hearing loss, and 2 had profound hearing loss. Postoperative hearing improvement was observed in patients 11-13, whereas no significant change was noted in patients 14-15. All 5 patients developed vertigo and tinnitus following the trauma. Among these, 2 patients (patients 11 and 13) exhibited incus dislocation, and 2 patients (patients 12 and 15) were diagnosed with pneumolabyrinth.

**Figure 2:**
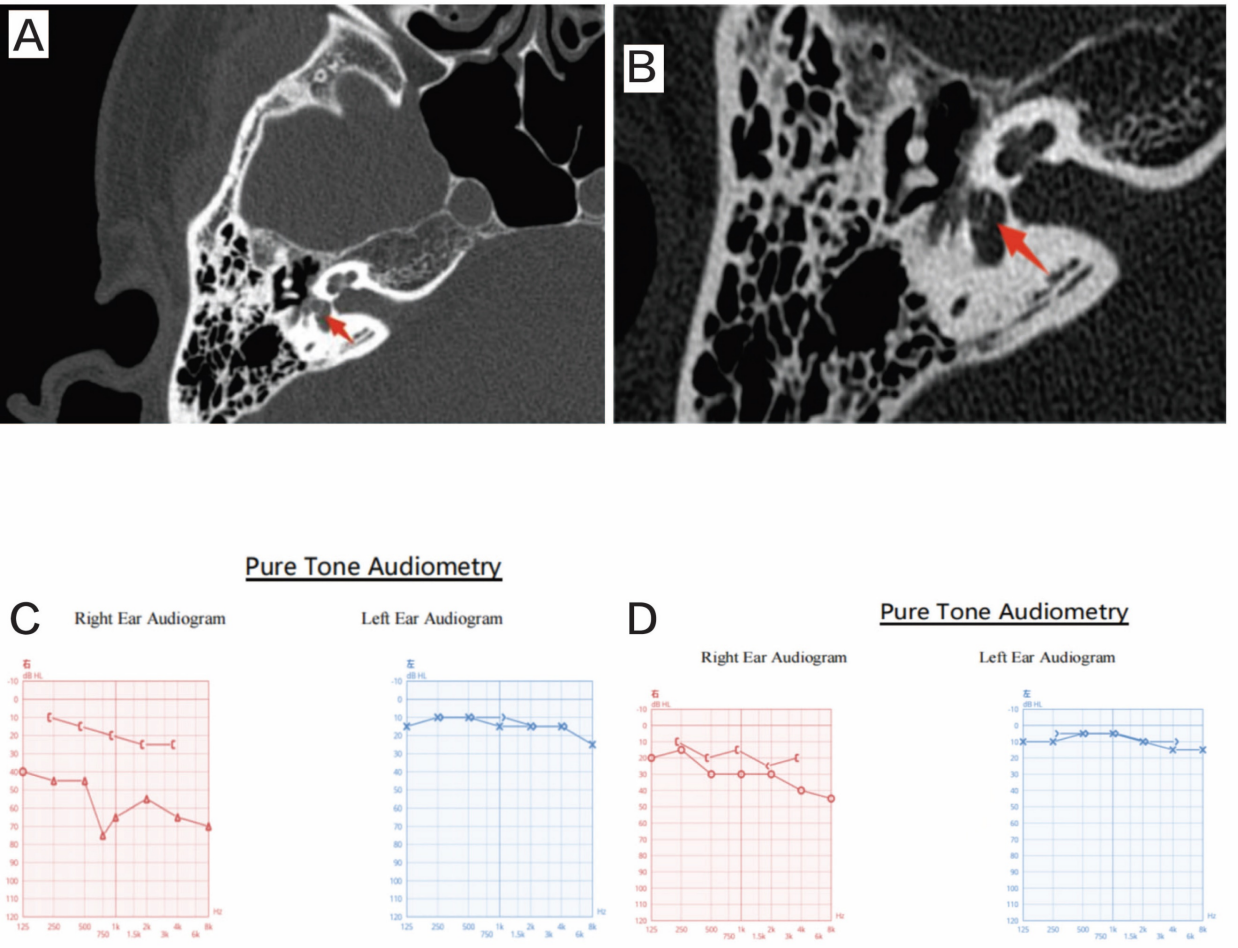
Case information for Patient 11. A and B: Stapediovestibular dislocation can be seen in the HRCT. C: the preoperative audiogram of patient 11. D: the postoperative audiogram.

In the 2 patients with Type II trauma and incus dislocation, intraoperative findings revealed depression of the stapes. Following the removal of the stapes, the incus dislocation was confirmed. The oval window was sealed with fat, and a piston (0.4 × 7 mm) was placed between the fat and the neck of the malleus. Postoperatively, vertigo resolved in these patients (Figure 2).

**Figure 3:**
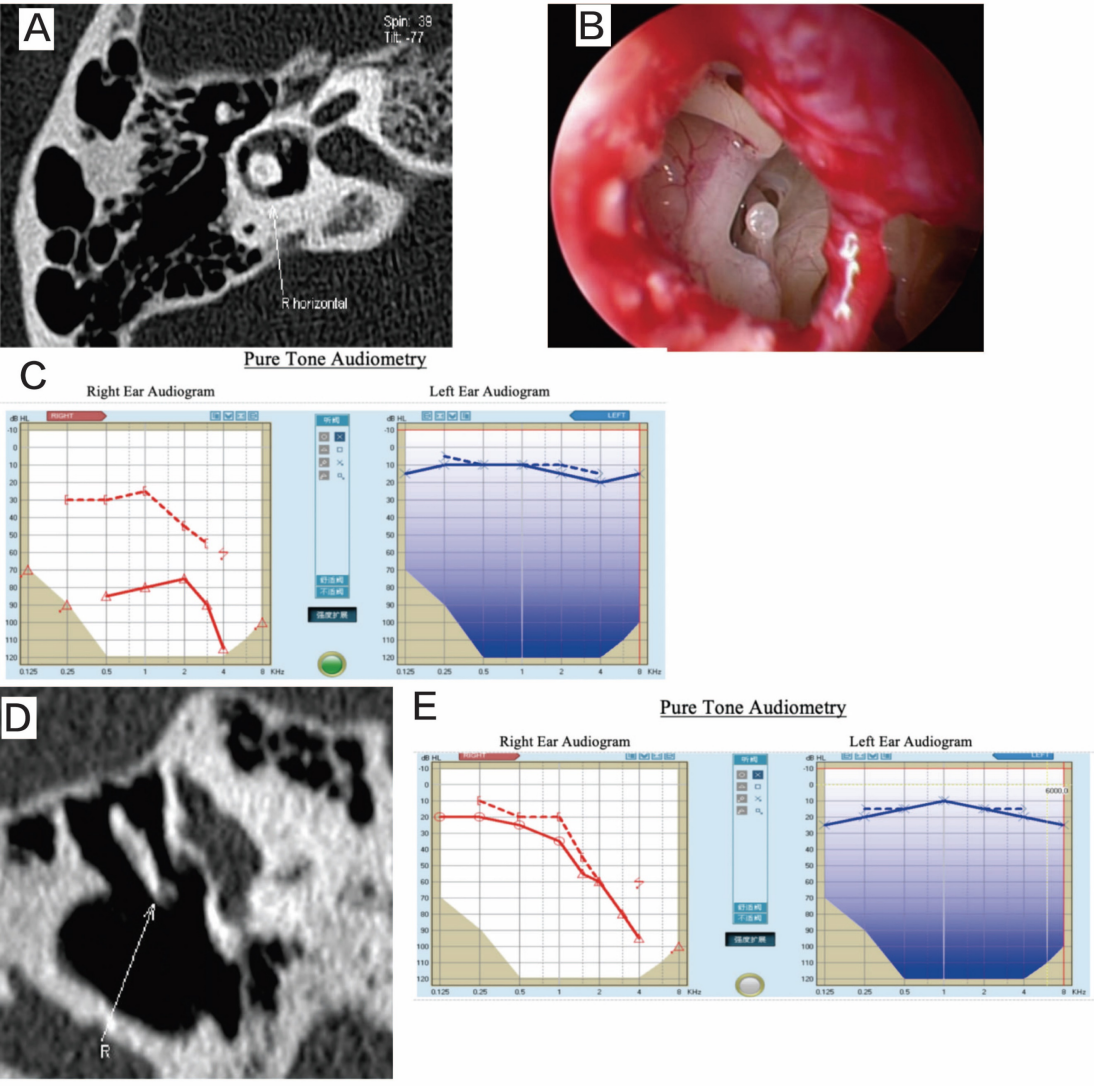
Patient 12. A: preoperative CT, revealing pneumolabyrinth. B: the deep depression of the stapes into the vestibule. C: preoperative audiogram. D: postoperative HRCT, showing that the pneumolabyrinth has been resolved. E: postoperative audiogram.

The 2 patients with pneumolabyrinth experienced recurrent positional vertigo and positive Roll test results after the trauma. Based on the trauma history and HRCT findings, it was determined that stapediovestibular dislocation with pneumolabyrinth had occurred. Intraoperative exploration revealed stapes depression. After removing the stapes, the vestibule was sealed with fat, and the stapes was repositioned. Vertigo resolved in both patients postoperatively (Figure 3).

**Figure 4:**
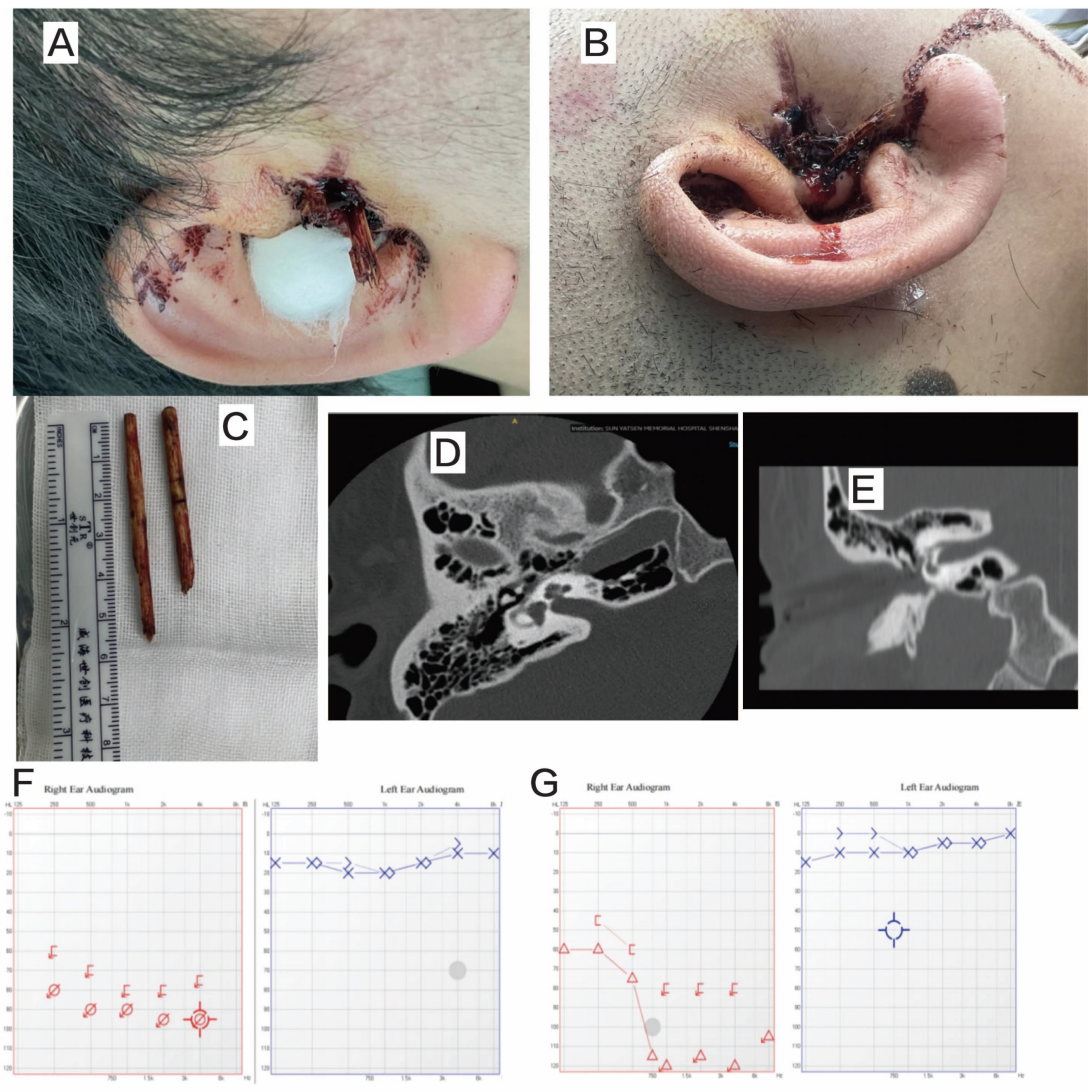
Patient 14. A and B: penetrating trauma to the external auditory canal (bamboo chopsticks). C: the foreign body (bamboo chopsticks). D and E: preoperative HRCT. F: preoperative audiogram. G: postoperative audiogram.

One patient was admitted to the emergency department on the day of the trauma. Pure tone audiometry indicated profound sensorineural deafness, and HRCT revealed incudomalleolar dislocation. Intraoperative exploration showed slight depression of the stapes with perilymphatic leakage from the oval window. The stapes was repositioned, and the footplate was sealed with fat. No perilymphatic fistulae were observed postoperatively, and the patient's vertigo resolved (Figure 4).

## Discussion

Ossicular chain trauma is predominantly caused by head injuries, while the incidence of direct injury (penetrating trauma) to the external auditory canal is relatively low 17, 18. A previous study reported that of 166 cases of traumatic ossicle dislocation, 160 (96%) were attributable to head trauma 3. Similarly, another study involving 42 patients with traumatic ossicle dislocation found that 39 (92.8%) had experienced head trauma 9. Interestingly, cultural practices appear to influence the etiology of such trauma. For instance, a study conducted in Japan identified ear-picking injuries as the most common cause of traumatic ossicle dislocation, which is linked to the cultural practice of using ear picks for ear cleaning in Japan 19, 20. In our country, the use of ear picks or cotton swabs for ear cleaning is also prevalent. In our study, 5 of the 15 patients sustained direct injuries to the external auditory canal: 3 from ear picking, 1 from an accidental stab with a tree branch, and 1 from the insertion of bamboo chopsticks into the ear canal.

Based on the presence of stapediovestibular dislocation, the traumas were categorized into Type I and Type II. Etiologically, Type I traumas were primarily caused by head injuries (9/10) 21, 22, while Type II traumas were exclusively associated with penetrating injuries to the external auditory canal (5/5). In terms of hearing impairment, Type I traumas were mainly accompanied by conductive hearing loss, whereas Type II traumas predominantly resulted in reduced bone conduction, with both mixed and profound sensorineural hearing loss being possible. Patients with Type II trauma exhibited more severe hearing impairment 21, while those with Type I trauma demonstrated more significant postoperative hearing improvement. Symptomatically, Type II patients were more likely to experience tinnitus and vertigo. We hypothesize that Type II patients suffered varying degrees of stapediovestibular dislocation, depression, perilymphatic fistula, or even pneumolabyrinth, which contributed to varying degrees of decreased bone conduction and vertigo. According to the literature, stapediovestibular dislocation can lead to lymphatic exposure, resulting in a range of cochlear vestibular symptoms, including sensorineural hearing loss, mixed hearing loss, and vertigo. Studies have indicated that some patients experience lymphadenopathy immediately following an accident. Patients who lose consciousness or sustain more serious injuries to other organs may overlook vestibular system symptoms. Regarding treatment delay, only 1 patient was admitted to the emergency department, while the remaining patients received conservative treatment at local hospitals and later sought further medical care due to hearing loss or vertigo. Compared to Type I trauma, the treatment delays for Type II trauma were shorter, likely due to the recurrent vertigo experienced by Type II patients after conservative treatment, whereas the longer treatment delay for Type I trauma may be attributed to the patients' primary concern with hearing improvement.

In treating Type I patients, ossiculoplasty was performed based on the separation or dislocation of the ossicles and the condition of the stapes. Partial ossicular replacement prostheses (PORPs) or total ossicular replacement prostheses (TORPs) were utilized for reconstruction. Postoperative hearing improvement was significant, with air-bone gap (ABG) thresholds reduced to within 10 dB in 50% (5/10) and within 20 dB in 100% (10/10) of patients. A previous study reported that 73.3% and 100% of patients had postoperative ABG thresholds within 10 dB and 20 dB, respectively 8. Another study 17 indicated that ABG thresholds ranged from 0-10 dB in 65% of patients and from 11-20 dB in 30%. Yet another study 23, 24 compared different ossicle materials and reconstruction methods, such as autologous ossicles or titanium ossicular replacement prostheses. In the study by Chien *et al.* 25, the hearing outcomes of the titanium prosthesis group and the autologous incus group were compared, and both groups demonstrated good hearing restoration.

For Type II patients, after intraoperative exploration of the vestibule and stapes, different ossicle reconstruction methods were employed depending on whether the incus was dislocated. In two cases of incus dislocation, both the stapes and incus were removed, the oval window was sealed with fat, and a piston (0.4 × 7 mm) was placed between the fat and the neck of the malleus. In two patients with normal incus anatomy but presenting with pneumolabyrinth, the stapes was first removed, the vestibule was filled with fat, and the stapes was repositioned between the incus and the fat. One patient with preoperative profound sensorineural deafness underwent stapes repositioning during surgery. However, hearing improvement was not significant in the two patients with profound sensorineural deafness, while the remaining three patients showed varying degrees of improvement 26.

Studies have shown that stapediovestibular dislocation is a rare occurrence 27-31, which can be categorized into internal dislocation (depression into the vestibule) and external dislocation, with the latter being even rarer. Internal dislocations are often caused by direct penetrating injuries with cotton swabs or ear picks, whereas external dislocation can occur when ligaments are torn by traumatic force. Comminuted fractures of the stapes footplate can also result in stapediovestibular dislocation. Surgical exploration in all five of our patients revealed internal dislocation 32, 33.

Currently, there is no consensus on the treatment protocol for stapediovestibular dislocation 34. Some studies suggest that removal of the stapes may cause additional damage, leading to postoperative hearing loss 28, 34, 35. It has also been proposed that if the stapes remains in the vestibule, scar formation around the stapes may eventually occupy the vestibular space, causing late-onset inner ear injury 36-38. Certain studies recommend surgical exploration to remove the stapes from the vestibule 28, 36, 39. Arragg and Paparella suggested that immediate surgical intervention, including resection or elevation of the stapes, is necessary for stapes fractures involving depression into the vestibule 30, 40. However, even when stapes depressed deep into the vestibule are removed, hearing restoration may not be satisfactory, and there is a risk of sensorineural hearing loss 34. According to one study 36, 41, when dislocation of the stapes into the vestibule is suspected, it is crucial to assess the depth of stapes depression and the presence of fractures. Studies have demonstrated that when the stapes are only slightly depressed into the vestibule, good hearing reconstruction outcomes can be achieved regardless of whether the stapes is resected 30, 42. However, when the stapes are deeply compressed into the vestibule, the risk of inner ear injury increases, leading to poor postoperative hearing recovery 37, 43, 44. The treatment of stapediovestibular dislocation depends on the integrity and position of the stapes, as well as the preoperative hearing status. The most common approaches include reduction or removal of the stapes, followed by sealing the oval window with fascia or fat before hearing reconstruction 45, 46.

In this study, two patients experienced recurrent paroxysmal positional vertigo after trauma, and pneumolabyrinth was detected via HRCT examination. Ederies *et al.* 47, 48 proposed the “pneumopositional” theory, wherein the movement of air bubbles in the vestibule or semicircular canal induces positional vertigo. This hypothesis is supported by HRCT findings 49, 50, which showed air movement in the vestibule or semicircular canal as the body position changed, leading to cupular deflection and ampullary stimulation, followed by the onset of nystagmus and vertigo. Tsubota *et al.* 51, 52 identified three predictive factors for hearing improvement following pneumolabyrinth: bone conduction hearing level after trauma, the interval between injury and surgery, and the presence of stapes lesions. A meta-analysis by Hidaka *et al.* 53, 54 found that while vestibular symptoms in patients with pneumolabyrinth were effectively alleviated after treatment, hearing outcomes varied: among those with pneumolabyrinth limited to the vestibular organ, 48% of patients experienced hearing improvement, but if pneumolabyrinth extended to both the vestibule and cochlea, hearing did not improve significantly. In our study, one patient with severe mixed hearing loss before surgery showed postoperative hearing improvement, while the other, who had very severe preoperative hearing loss, did not experience hearing improvement after surgery. The meta-analysis by Hidaka *et al.* found no significant difference in efficacy between conservative and surgical treatments 55, 56.

In our study, patients with Type I trauma showed substantial postoperative hearing improvement. Different ossiculoplasty methods were selected based on the presence of structures above the stapes 57, 58. Ossicular replacement prostheses were used for reconstruction, resulting in effective postoperative hearing improvement. Conversely, in Type II patients who experienced repeated paroxysmal positional vertigo after the trauma event, pneumolabyrinth should be suspected. Postoperatively, vertigo in Type II patients was effectively alleviated, although hearing compensation was poor 59, 60. After carefully removing the stapes during surgery, the vestibule was sealed with fat or fascia 61, 62. Different hearing reconstruction methods were chosen based on the condition of the incus. If the incus was normal, the stapes were reduced; if the incus was dislocated, a piston (0.4 × 7 mm) was used for hearing reconstruction. Treatment strategies were tailored according to the classification, guiding the prognosis of the patients 63.

The sample size of this study was small, and future research should aim to increase the number of patients analyzed. Additionally, for Type I patients, a control group consisting of those undergoing different ossiculoplasty methods, such as autologous ossicles, could be included. Extending the follow-up period would allow for better observation of patient prognosis, particularly in terms of vertigo and hearing improvement in Type II trauma patients.

## Funding

This research is supported by the National Natural Science Foundation of China (Grant No. 82101227) and the Science and Technology Foundation of Guangzhou (Grant No. 2023A04J2091).

## Figures and Tables

**Table 1:** Clinical Characters of 15 Cases with Traumatic Ossicular Chain Dislocation

Patient	Age/Sex	Trauma etiology	Treatment delay	Clinical symptoms	Intraoperative finding	Management	Audiometric examination	Preop-PTA, dB	Preop-ABG, dB	Postop-PTA, dB	Postop-ABG, dB
1	14/M	Head trauma	1month	Hearing loss	IS separation	PORP	Conductive	50	35	30	12.5
2	40/M	Head trauma	1month	Hearing loss	IS separation	PORP	Conductive	58.75	35	33.75	10
3	51/M	Head trauma	1month	Hearing loss	Incus dislocation	PORP	Conductive	48.75	37.5	20	7.5
4	62/F	Ear-pick	13days	Hearing loss, Tinnitus	Incus dislocation Fracture of the anterior and posterior arch of the stape	TORP	Mixed	92	45	60	15
5	31/M	Head trauma	9months	Hearing loss	IM separation	PORP	Conductive	44	31	28	12
6	26/M	Traffic accident	2years	Hearing loss, Tinnitus	IS separation	PORP	Mixed	76	32.5	40	10
7	38/M	Head trauma	7months	Hearing loss, Tinnitus	IM separation	PORP	Mixed	82	40	45	9
8	41/F	Head trauma	1month	Hearing loss	IS separation	PORP	Conductive	47	35	24	9
9	49/F	Head trauma	3years	Hearing loss	IS separation	PORP	Conductive	65	42	35	11
10	31/F	Head trauma	6years	Hearing loss, Tinnitus	Fracture of the anterior and posterior arch of the stape	TORP	Conductive	67	40	36	12
11	39/M	The branch	3months	Hearing loss, Vertigo, Tinnitus	Incus dislocation, Stapediovestibular Dislocation	Piston (0.4*7mm)	Conductive	57.5	36.25	32.5	12.5
12	42/M	Ear-pick	5months	Hearing loss, Vertigo, Tinnitus	Stapediovestibular Dislocation pneumolabyrinth	RO	Mixed	88.75	48.75	53.75	5
13	33/M	Ear-pick	1year	Hearing loss, Vertigo, Tinnitus	Incus dislocation, Stapediovestibular Dislocation	Piston (0.4*7mm)	Mixed	85	30	70	25
14	19/M	Bamboo chopsticks	Immediately	Hearing loss, Vertigo, Tinnitus	IS separation, Stapediovestibular Dislocation	Medication and RO	Profound sensorineural	>100	NA	>100	NA
15	50/M	Ear-pick	4months	Hearing loss, Vertigo, Tinnitus	Stapediovestibular Dislocation pneumolabyrinth	Medication and RO	Profound sensorineural	>100	NA	>100	NA

RO, restoration of the stapes to its anatomical place

**Table 2:** The Classification for Traumatic Ossicular Chain Dislocation

	Type I	Type II
Total number of patients	10	5
Cause: Head trauma (impact, car accident)	9	/
Cause: Penetrating trauma of the external auditory canal	1	5
Audiometric examination		
Conductive	7	1
Mixed	3	2
Profound sensorineural	/	2
